# Genotypic comparison of *Pantoea agglomerans *plant and clinical strains

**DOI:** 10.1186/1471-2180-9-204

**Published:** 2009-09-22

**Authors:** Fabio Rezzonico, Theo HM Smits, Emilio Montesinos, Jürg E Frey, Brion Duffy

**Affiliations:** 1 Agroscope Changins-Wädenswil ACW, Plant Protection Division, CH-8820 Wädenswil, Switzerland; 2 Institute of Food and Agricultural Technology-CIDSAV-XaRTA, University of Girona, E-17071 Girona, Spain

## Abstract

**Background:**

*Pantoea agglomerans *strains are among the most promising biocontrol agents for a variety of bacterial and fungal plant diseases, particularly fire blight of apple and pear. However, commercial registration of *P. agglomerans *biocontrol products is hampered because this species is currently listed as a biosafety level 2 (BL2) organism due to clinical reports as an opportunistic human pathogen. This study compares plant-origin and clinical strains in a search for discriminating genotypic/phenotypic markers using multi-locus phylogenetic analysis and fluorescent amplified fragment length polymorphisms (fAFLP) fingerprinting.

**Results:**

Majority of the clinical isolates from culture collections were found to be improperly designated as *P. agglomerans *after sequence analysis. The frequent taxonomic rearrangements underwent by the *Enterobacter agglomerans/Erwinia herbicola *complex may be a major problem in assessing clinical associations within *P. agglomerans*. In the *P. agglomerans sensu stricto *(in the stricter sense) group, there was no discrete clustering of clinical/biocontrol strains and no marker was identified that was uniquely associated to clinical strains. A putative biocontrol-specific fAFLP marker was identified only in biocontrol strains. The partial ORF located in this band corresponded to an ABC transporter that was found in all *P. agglomerans *strains.

**Conclusion:**

Taxonomic mischaracterization was identified as a major problem with *P. agglomerans*, and current techniques removed a majority of clinical strains from this species. Although clear discrimination between *P. agglomerans *plant and clinical strains was not obtained with phylogenetic analysis, a single marker characteristic of biocontrol strains was identified which may be of use in strain biosafety determinations. In addition, the lack of Koch's postulate fulfilment, rare retention of clinical strains for subsequent confirmation, and the polymicrobial nature of *P. agglomerans *clinical reports should be considered in biosafety assessment of beneficial strains in this species.

## Background

*Pantoea agglomerans *(Beijerinck 1888) comb. nov. [1], formerly *Enterobacter agglomeran*s (Beijerinck 1888) Ewing and Fife (1972), *Erwinia herbicola *(Löhnis 1911) Dye 1964 or *Erwinia milletiae *(Kawakami and Yoshida 1920) Magrou 1937, is a Gram-negative bacterium that belongs to the family of Enterobacteriaceae. *P. agglomerans *is primarily a plant epiphyte [2-4] commonly found in diverse ecological niches including aquatic environments, soil or sediments [5-7]. Several strains of *P. agglomerans *are sold as commercial biological control agents (BCAs) against the fire blight pathogen [*Erwinia amylovora *(Burrill 1882) Winslow et al. 1999] on apple and pear trees [8,9]. *P. agglomerans *strains are effective against other bacterioses, such as basal kernel blight of barley [10] and post-harvest fungal diseases of pome fruits [11-14]. Three commercial *P. agglomerans *strains have recently been registered for biocontrol of fire blight in New Zealand (BlossomBless™ strain P10c [15]), in the United States and in Canada (BlightBan C9-1™ strain C9-1 [16]; Bloomtime™ strain E325 [17]). The primary mode of action is competitive exclusion which involves the occupation of sites otherwise colonized by the pathogen, but for some strains reports also indicate the contribution of different antibiotics like herbicolins [16] pantocins [18-21], putatively phenazine [22], and other unknown compounds [17].

Despite efficacy trials in commercial orchards demonstrating the potential of *P. agglomerans *biocontrol formulations as an alternative plant protection tool and their approval in the United States by the Environmental Protection Agency (EPA) as microbial pesticides http://www.epa.gov/fedrgstr/EPA-PEST/2006/September/Day-20/p8005.htm, registration efforts in Europe are hindered by biosafety concerns stemming from clinical reports that identify strains of *P. agglomerans *as opportunistic human pathogens, and have resulted in the current classification of this species as a biosafety level 2 (BL-2) organism in Europe [23-27]. Biosafety classification differs among countries; in the European Union, Directive 2000/54/EC includes "*Enterobacter *spp." in the list of microorganisms that are currently classified as a biosafety level 2 (BL-2), while the German "Technische Regeln für Biologische Arbeitsstoffe", TRBA 466 and Swiss regulations http://www.bafu.admin.ch/publikationen/publikation/00594/index.html?lang=de more explicitly identify *P. agglomerans *and its synonyms in BL-2. Several strains maintained in culture collections throughout the world and the type strain *P. agglomerans *LMG 1286^T ^(= CDC 1461-61^T ^= NCTC 9381^T ^= ICMP 3435^T ^= ATCC 27155^T^) itself are listed as clinical isolates [1]. Confirmed pathogenicity of this species is difficult to ascertain, since clinical reports involving *P. agglomerans *are typically of polymicrobial nature, often involve patients that are already affected by diseases of other origin, lack Koch's postulate fulfillment or any pathogenicity confirmation, and diagnostic isolates are rarely conserved for confirmatory analysis [24].

There has been insufficient investigations as to whether agriculturally beneficial isolates are distinct from clinical isolates or harbor potential pathogenic determinants that would justify current biosafety restrictions. Comparative analysis of biocontrol and clinical strains, or examination of virulence factors has been performed previously for other 'Jekyll-Hyde' biocontrol species reported to contain opportunistic pathogenic isolates, such as *Pseudomonas aeruginosa *[28-30], *Serratia marcescens *[31] and the *Burkholderia cepacia *complex [32,33]. Similar comparisons have not been performed for *P. agglomerans*, leaving a gap in knowledge critical to regulatory authorities.

The aim of our study was to perform a polyphasic genotypic and phenotypic analysis of *P. agglomerans *isolates of diverse origin in order to understand whether clinical and biocontrol (environmental) isolates can be distinguished and have undergone a discrete evolution that would indicate specialization towards human pathogenicity or an epiphytic lifestyle. The taxonomy of a collection of clinical and plant isolates was assessed using fluorescent amplified fragment length polymorphism (fAFLP) analysis of total genomic DNA and sequence analyses of specific genes (such as 16S rDNA gene *rrs*, *gyrB *encoding DNA gyrase subunit B, and the *P. agglomerans *quorum-sensing regulatory genes *pagRI *encoding homoserine lactone receptor and synthase) [34]. The fAFLP analysis was used as well to search for random molecular markers that could serve as a simple and rapid discriminatory marker for clinical and biocontrol strains. Additionally, we examined the distribution of some phenotypic and genotypic traits among strains that may reflect adaptation to the different lifestyles proposed for *P. agglomerans*, such as growth at 37°C for clinical isolates, presence of pantocin A genes or sorbitol utilization for biocontrol strains, and presence of type III secretion system (T3SS) for plant pathogenic pathovars.

## Methods

### Bacterial strains

Thirty-two clinical isolates designated as *P. agglomerans*, *E. agglomerans*, *E. herbicola *or *Pantoea *spp. were obtained from the American Type Culture Collection (ATCC, http://www.atcc.org/), the Belgian Coordinated Collection of Microorganisms (BCCM/LMG, http://bccm.belspo.be), the Institut Pasteur Collection (CIP, http://www.crbip.pasteur.fr/), the Spanish Type Culture Collection (CECT, http://www.cect.org/) or received from the Hospital de la Santa Crei Sant Pau (Barcelona, Spain) and the Istituto Cantonale di Microbiologia (ICM, Bellinzona, Switzerland). Eleven *P. agglomerans *strains with established biocontrol activity obtained from several sources (including the three currently registered commercial strains), twenty environmental isolates and three phytopathogenic strains, together with representative strains of other *Pantoea *species and closely related genera such as *Erwinia*, *Pectobacterium *and *Brenneria*, were included in the study for comparison (see Additional file 1 - Table S1).

### DNA extraction and PCR amplification

DNA of each bacterial isolate was extracted with the Wizard^® ^Genomic DNA Purification Kit (Promega, Dübendorf, Switzerland) from 1.5 ml aliquots of overnight cultures at 28°C in Luria Bertani (LB) medium. Obtained genomic DNA was quantified on a NanoDrop 1000 spectrophotometer (Thermo Scientific, Wilmington, U.S.A.) and 10-20 ng of genomic DNA were used for each PCR reaction. Each PCR was performed in a total volume of 25 μl using 0.4 mM of each primer in a final concentration of 1× master mix of the HotStarTaq Master Mix Kit (Qiagen, Basel, Switzerland). PCR targeting the 16S rRNA gene *rrs*, *gyrB *housekeeping gene, *pagRI *AHL receptor and synthase genes, T3SS ATPase *hrcN*, and the insertion site of the *P. agglomerans *genomic island carrying the pantocin genes *paaABC *and these genes were performed. Primer sequences and annealing temperature (T_m_) for each PCR are shown in Table 1. With the exception of the *gyrB *amplification standard cycling conditions were used for all PCRs with an initial denaturation and activation of the HotStarTaq enzyme for 15 min at 95°C, followed by 35 cycles of denaturation at 95°C for 30 s, annealing at the proper T_m _for 45 s, plus 30 s of elongation at 72°C for every 500 bp of expected amplicon size, ending with a final elongation for 10 min at 72°C. The protocol for *gyrB *amplification included, after the initial polymerase activation, 42 cycles of denaturation at 95°C for 30 s, 30 s annealing at 50°C where the annealing time increased by 2 s/cycle until 40 s were reached, plus 10 s elongation at 72°C where the extension time increased by 1 s/cycle until 15 s are reached. Positive PCR amplification was verified by loading 5 μl of each reaction on a 1.2% agarose gel.

**Table 1:** PCR primers used for gene amplification and sequencing.

Gene(s)	Primer name	Sequence (5'-3')	Size (bp)	Tm (°C)	Reference
*gyrB*	gyr-320	TAARTTYGAYGAYAACTCYTAYAAAGT	970	50	[63]
	rgyr-1260	CMCCYTCCACCARGTAMAGTTC			[63]
*hrcN*	hrcN-4r	CGAGCAGGAYTCGATGAACG	250	50	[57]
	hrcN-5rR	CCGGWYTGGTATTCACCCAG			[57]
29-kbp GI	mutS-rev	CGCCATCGGGATCGGTTCGCC	554	60	This work
	narL-rev	GCCGTCTGGGCGCTGCAGAACG			This work
*paaABC*	paaA-fw	CTCTTGCCAAAATGGATGGT	2398	55	This work
	paaC-rev	TTGCAAATTCTGCACTCTCG			This work
*pagRI*	pagR-fw	GTGAAGGATACYTACTACAACG	1206-29	55	This work
	pagI-rev	CGAATGCATTGACGGCATGG			This work
*rrs*	16S-8F	AGAGTTTGATCCTGGCTCAG	1503	48	[64]
	16S-533R	TTACCGCGGCTGCTGGCAC			[64]
	16S-609R	ACTACYVGGGTATCTAAKCC			[65]
	16S-1492R	ACGGTTACCTTGTTACGACTT			[64]

Tm = annealing temperature

### Sequencing of 16S rDNA, gyrB and pagRI genes

PCR amplicons were purified from the PCR mix by washing twice with 50 μl of double-distilled water (ddH_2_O) on a MultiScreen PCR Plate (Millipore, Molsheim, France), resuspended in 30 μl of ddH_2_O, and quantified spectrophotometrically as described above. The cycle-sequencing reaction was performed with 20-40 ng of purified PCR product using the ABI PRISM BigDye Terminators v1.1 Cycle Sequencing Kit (Applied Biosystems, Foster City, CA, U.S.A.) according to the manufacturer instructions employing the same primers used for PCR amplification. For 16S rRNA gene sequencing, additional primers 16S-609R and 16S-533R (Table 1) were used to obtain complete coverage of the amplicon. Cycle sequencing products were cleaned through water-swelled Sephadex G-50 columns (Amersham Biosciences, Uppsala, Sweden) on MultiScreen HV plates (Millipore) and sequenced on an ABI PRISM 3100 Genetic Analyzer. Obtained sequences were assembled using the Sequencher software (version 4.0.5; Gene Codes Corporation, Ann Arbor, MI, U.S.A.).

### Phylogenetic analysis of sequencing data

Phylogenetic trees were generated on the basis of partial 16S rDNA, *gyrB *and *pagRI *sequences without choosing any outgroup. DNA sequences were aligned with ClustalW [35]. Sites presenting alignment gaps were excluded from analysis. The Molecular Evolutionary Genetics Analysis (MEGA) program version 4.0 [36] was used to calculate evolutionary distances and to infer trees based on the Minimum Evolution (ME) method using the Maximum Composite Likelihood (MCL) formula. Nodal robustness of the inferred trees was assessed by 1000-bootstrap replicates.

### Identification of non-Pantoea strains

For those strains received as *E. agglomerans*, *P. agglomerans *or *Pantoea *spp. from international culture collections but not clustering with *P. agglomerans *in the 16S rDNA and *gyrB *trees, identification was sought by blasting the obtained nucleotide sequences in the NCBI database. Since the best hits often led to poorly characterized or obviously misdentified bacteria only the best match with a secure identification was retained. Confidence of secure identifications was based either on relatedness to the *P. agglomerans *type strain or position in the BLAST distance tree. In order to be considered trustworthy, obtained hits were required to be flanked by sequences of representatives of the same species and not be part of a clade containing strains from related species with dissimilar identification.

### fAFLP analysis

The fAFLP pattern of strains identified by sequencing as *P. agglomerans sensu stricto *(in the stricter sense taxonomically) was carried out following standard protocols with minor modification [37-39]. Digestion of genomic DNA and ligation to the restriction enzyme adaptors was performed simultaneously since a base-change incorporated into the adaptors sequences hindered restoration of the original restriction enzyme site upon ligation. Between 200-400 ng genomic DNA from each of the strains was used for each reaction in a mix containing 5 units EcoRI (Roche, Basel, Switzerland), 1 unit of MseI (Roche) and 1 unit of T4 DNA Ligase (Epicentre, Madison, U.S.A.), 5 mM 1,4-Dithio-DL-threitol (DTT) (Sigma-Aldrich, Buchs, Switzerland), 200 μM ATP (Fermentas, St. Leon-Rot, Germany), 50 μg/ml Bovine Serum Albumin (BSA), 0.25 μM of each EcoRI adaptor (EcoRI-F 5'-CTCGTAGACTGCGTACC-3', EcoRI-R 5'-AATTGGTACGCAGTCTAC-3') and 2.5 μM of each MseI adaptor (MseI-F 5'-GACGATGAGTCCTGAG-3', MseI-R 5'-TACTCAGGACTCAT-3') in a total volume of 11.1 μl of 1× One-Phor-All Buffer PLUS (GE Healthcare, Otelfingen, Switzerland). The reaction was incubated for 3 h at 37°C and then heated for 15 min at 72°C. The digestion-ligation mixture was diluted 1:20 with nuclease-free water and 5 μl of the dilution were pre-amplified in a total volume of 20 μl with adaptor-specific primers MseI-00 (5'-GATGAGTCCTGAGTAA-3') and EcoRI-00 (5'-GACTGCGTACCAATTC-3'). The PCR mix contained 1 unit Taq DNA polymerase (Promega), 0.2 mM dNTPs and 0.3 μM of each primer in 1× Taq DNA polymerase buffer (MgCl_2 _1.5 mM) (Promega). The PCR conditions consisted of an initial incubation for 5 min at 72°C to allow Taq DNA polymerase to fill the nick on the ligated DNA strand, followed by 20 cycles of denaturation at 94°C for 30 s, annealing at 56°C for 30 s, plus 2 min of elongation at 72°C, finishing with a final extension for 2 min at 72°C and 30 min incubation at 60°C. Each pre-amplification reaction was diluted 1:10 with nuclease-free water and 5 μl of each dilution were used in the selective amplification reactions, which were performed in a total volume of 20 μl using one selective primer (0.3 μM) for each restriction site in a final concentration of 1× HotStarTaq Master Mix Kit. The combinations of selective primers used are listed in Table 2. Cycling conditions consisted of an initial denaturation/activation at 94°C for 15 min and 20 cycles of denaturation at 94°C for 30 s, annealing at 66°C for 30 s where the annealing temperaure was decreased by 1°C/cycle until 56°C were reached, plus 2 min elongation at 72°C, followed by a final incubation at 60°C for 10 min. The PCR product was finally diluted 1:250 with nuclease-free water and 5 μl of each dilution were used in the labelling reaction, which was carried out under the same conditions as the selective amplification reactions except for the substitution of the EcoRI+1 selective primer with a FAM fluorophor 5'-labelled EcoRI+00 (no selective nucleotides) primer. One microliter of each reaction was mixed with 15 μl formamide containing 0.25 μl of LIZ500 standard (Applied Biosystems), denaturated for 10 min at 95°C and loaded on an ABI 3130XL sequencer (Applied Biosystems) for fAFLP fragment separation.

**Table 2:** Primers combinations and number of different peak positions generated used in the selective amplification step.

Name	Primer I	Sequence (5'-3')	Primer II	Sequence (5'-3')	# Peaks
AT	EcoRI-A	GACTGCGTACCAATTC**A**	MseI-T	GATGAGTCCTGAGTAA**T**	250
CGC	EcoRI-C	GACTGCGTACCAATTC**C**	MseI-GC	GATGAGTCCTGAGTAA**GC**	202
TG	EcoRI-T	GACTGCGTACCAATTC**T**	MseI-G	GATGAGTCCTGAGTAA**G**	183
GG	EcoRI-G	GACTGCGTACCAATTC**G**	MseI-G	GATGAGTCCTGAGTAA**G**	250

Selective bases are in boldface.

### Analysis of fAFLP data

Raw data collected from the ABI 3130XL sequencer were analyzed using the GeneMapper v4.0 software (Applied Biosystems). To remove noise, only peaks with an absolute intensity greater than 200 (combinations CGC, TG) or 300 (combinations GG, AT) were retained for final analysis. The fAFLP profiles were converted into a binary matrix of presence/absence of each peak and this data was used to construct a UPGMA (Unweighted Pair Group Method with Arithmetic mean) dendrogram using the MEGA software. Nodal robustness of the inferred trees was assessed by 1000-bootstrap replicates. The fAFLP patterns of all strains were analyzed in order to identify peaks that may be distinctive of either biocontrol or clinical isolates in agreement with the classification shown in Additional file 1 - Table S1.

### Growth on sorbitol as sole carbon source

Growth ability of *P. agglomerans *strains on sorbitol was studied using 200-μl microcultures in 100-well Bioscreen C MBR system honeycomb plates (well volume 400 μl) at 24°C with regular shaking at 15-min intervals in M9 minimal medium containing 10 mM sorbitol as sole carbon source. All strains were grown overnight in LB, collected by centrifugation, and washed twice with sterile 0.9% NaCl before being inoculated in M9 at an initial OD_600 _of about 0.02. Growth curves were measured in triplicates by periodically quantifying the absorbance through a 420- to 580-nm wide band filter (OD_420-580 nm_) using a Bioscreen C MBR system (Growth Curves Oy, Helsinki, Finland).

### Growth at 24°C and 37°C

Growth ability of selected *P. agglomerans sensu stricto *strains was determined at 24°C and 37°C using the Bioscreen C MBR system. The protocol was the similar to that described above for growth on sorbitol, except that LB medium was used in place of minimal medium. The mean growth rate per hour (*k*) was calculated each 20 minutes according to the formula

where *N*_0 _and *N*_*t *_represent absorbance measured at two consecutive time points and Δ*t *is the time interval (i.e., 1 h) between the two measurements. The highest optical density, the maximal growth rate, as well as the time needed to reach the latter value were recorded for each strain. A comparison of these parameters was performed among the average values obtained for clinical, biocontrol or plant-pathogenic *P. agglomerans *strains. Correlations between OD_420-580 nm _measured in the Bioscreen C MBR system and number of colony forming unit (CFU) was estimated for representative strains by dilution plating on LB agar.

### Accession numbers

The accession numbers for the sequences produced for this study are: 16S rRNA gene [GenBank: FJ611802-FJ611887]; *gyrB *gene [GenBank: FJ617346-FJ617427]; *hrcN *gene [GenBank: FJ617428-FJ617436]; *pagRI *genes [GenBank: FJ656221-FJ656252].

With the exception of *pagRI*, for which they are shown directly in the corresponding figure, accession numbers and other sources of reference sequences not obtained in this work are indicated below. **Complete genomes**: *C. koseri *ATCC BAA-895 [NCBI: NC_009792], *E. amylovora *Ea273 http://www.sanger.ac.uk/Projects/E_amylovora/, *E. coli *K-12 MG1655 [NCBI: NC_000913], *Enterobacter *sp. 638 [NCBI: NC_009436], *E. tasmaniensis *Et1/99 [NCBI: NC_010694], *K. pneumoniae *342 [NCBI: NC_011283], *P. stewartii *subsp. *indologenes *DC283 http://www.hgsc.bcm.tmc.edu/microbial-detail.xsp?project_id=125. **16S rRNA gene**: *E. cloacae *ATCC 13047^T ^[GenBank: AJ251469], *E. sakazakii *ATCC 51329 [GenBank: AY752937], *Pantoea sp. *LMG 2558 [GenBank: EF688010], *Pantoea sp. *LMG 2781 [GenBank: EU216736], *Pantoea sp. *LMG 24198 [GenBank: EF688009], *Pantoea sp. *LMG 24199 [GenBank: EF688012], *Pantoea sp. *LMG 24200 [GenBank: EF688011], *Pantoea sp. *LMG 24534 [GenBank: EU216737], *P. terrea *LMG 22051^T ^[GenBank: EF688007], *S. enterica *sv *typhi *CT18 [NCBI: NC_003198]. ***gyrB *gene**: *E. cloacae *ATCC 13047^T ^[GenBank:EU643470], *E. sakazakii *ATCC 51329 [GenBank:AY370844], *Pantoea *sp. BD502 [GenBank: EF988786], *Pantoea *sp. BCC757 [GenBank: EF988776], *Pantoea sp. *LMG 2558 [GenBank: EF988812], *Pantoea sp. *LMG 2781 [GenBank:EU145271], *Pantoea sp. *LMG 24196 [GenBank: EF988758], *Pantoea sp. *LMG 24199 [GenBank: EF988768], *Pantoea sp. *LMG 24200 [GenBank: EF988770], *Pantoea sp. *LMG 24202 [GenBank: EF988778], *Pantoea sp. *LMG 24534 [GenBank: EU145269], *P. terrea *LMG 22051^T ^[GenBank:EF988804], *S. enterica *sv *typhi *CT18 [NCBI: NC_003198].

## Results

### PCR amplification and sequencing of 16S rDNA, gyrB and pagRI genes

**Figure 1:**
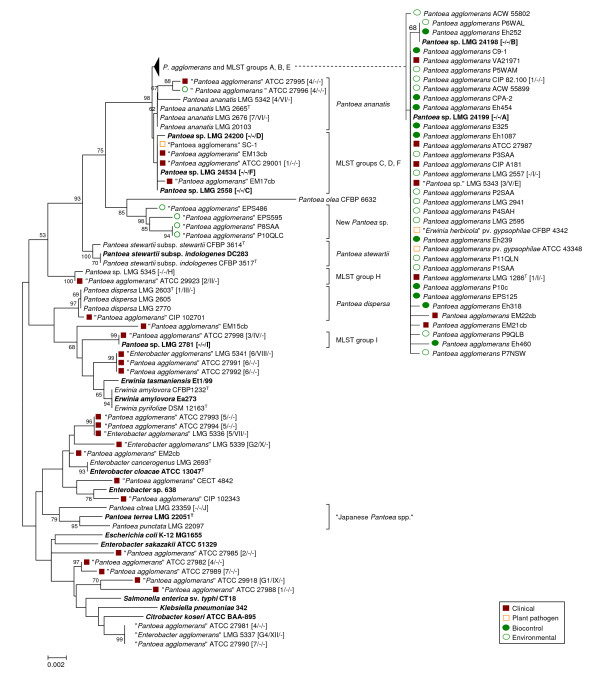
**Taxonomy of clinical, biocontrol, plant pathogenic and environmental isolates received as P. agglomerans, E. agglomerans, E. herbicola or Pantoea spp. based on 16S rDNA sequences**. The trees were constructed with the Minimum Evolution method using a 1338-bp fragment of the *rrs *gene (1235 positions, gaps completely removed from the analysis). Nodal supports were assessed by 1000-bootstrap replicates. Only bootstrap values greater than 50% are shown. The scale bar represents the number of base substitutions per site. Reference strains are marked in bold (^T ^= type strain). Where available the classification in biogroups [50], biotypes [41] and MLST-groups [40] is indicated between brackets. For improved clarity, the branch embracing *P. agglomerans *and MLST groups A, B and E was compressed in the main tree and is shown expanded on the right side of the figure.

**Figure 2:**
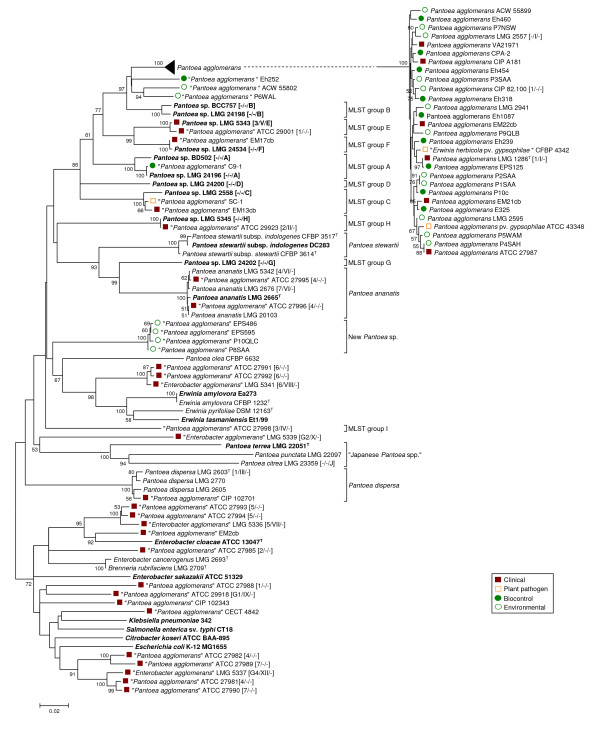
**Taxonomy of clinical and biocontrol isolates received as P. agglomerans, E. agglomerans or Pantoea spp. based on gyrB gene sequences**. The trees were constructed with the Minimum Evolution method using a 747-bp fragment of the gene (725 positions, gaps completely removed from the analysis). Nodal supports were assessed by 1000-bootstrap replicates. Only bootstrap values greater than 50% are shown. The scale bar represents the number of base substitutions per site. Reference strains are marked in bold (^T ^= type strain), for strains retrieved in a culture collection the classification in biogroups [50] and biotypes [41] and MLST-groups [40] is indicated between brackets.

Both 16S rDNA and *gyrB *primer sets were able to amplify the related fragments in all of the strains tested, wheras PCR amplification of *pagRI *genes was only successful for those strains which according to 16S rDNA and *gyrB *phylogenies were closely related to *P. agglomerans *type strain LMG 1286^T ^(Figure 1 &2). The use of primer 16S-8F for forward sequencing of the *rrs *gene proved challenging for many strains, especially those belonging to *P. agglomerans sensu stricto*, since the peaks on the electropherogram were frequently superimposed at the very beginning of the read making base calls virtually impossible. Independent sequencing of all seven 16S rDNA genes found *P. agglomerans *C9-1 revealed insertions of guanidine at position 80 and cytosine at position 90 in four copies of the gene, which resulted in a frameshift in the remainder of the gene sequence. Only reverse primers were utilized to sequence *rrs *with the final 90 bp discarded from subsequent analysis of the complete strain collection.

### Taxonomy of clinical and biocontrol P. agglomerans isolates

Sequence analysis of *gyrB *revealed that 26 of the 32 clinical isolates obtained from international culture collections as *P. agglomerans*, *E. agglomerans *or *Pantoea *spp. did not justifiably belong to *P. agglomerans*, but clustered distant from type strain LMG 1286^T^. Based on genotypic similarity, these strains belonged either to other *Pantoea *spp. or other Enterobacteriaceae genera. In contrast, classification of biocontrol strains was more precise than for presumptively clinical strains, and all of these could be identified unequivocally as *P. agglomerans sensu stricto *(Figure 2).

Congruence between phylogenies derived from *rrs *(Figure 1) and *gyrB *(Figure 2) gene sequences was imperfect. Analysis using 16S rDNA enabled only limited separation of strains within each *Pantoea *spp., whereas analysis using *gyrB *sequences revealed higher variability and enabled finer resolution of distinct branches with some strains clustering alongside *P. agglomerans *LMG 1286^T ^in the *rrs *tree. The *gyrB *clades corresponded largely to the MLST-groups recently defined by Brady et al. [40] for *Pantoea *spp. (Figure 2).

Four strains (EM13cb, EM17cb, ATCC 29001 and SC-1) that grouped with representative strains of *Pantoea *MLST-groups C, D and F in the *rrs *tree clearly diverged using *gyrB *sequences. Clinical isolate EM13cb and cotton pathogen SC-1 clustered with LMG 2558 (MLST-group C), while two other clinical isolates, EM17cb and ATCC 29001, clustered with LMG 24534 (MLST-group F) and LMG 5343 (MLST-group E) using either *rrs *or *gyrB*. In contrast, LMG 5343, LMG 24198 (MLST-group B) and LMG 24199 (MLST-group A), all clustered unexpectedly with *P. agglomerans *in the *rrs *tree (Figure 1) but were clearly divergent using *gyrB*. This demonstrated the resolution limits of 16S rDNA sequence analysis among *Pantoea *spp. Both *rrs *and *gyrB *sequences assigned two additional presumptive-clinical strains (ATCC 27995 and ATCC 27996) to the related species *Pantoea ananatis *(Serrano 1928) Mergaert et al. 1993, while most of the other human isolates (including representatives from Brenner's biotypes VII-XII [41]) clustered far from the *P. agglomerans sensu stricto *group and could be roughly assigned to *Erwinia *or *Enterobacter *spp. (see Additional file 2 - Table S2) based on BLAST comparison. Indicative of the uncertainty surrounding identification of this species, the BLAST best-hits list often included isolates clearly misidentified as *P. agglomerans *or *Pantoea *spp. Specifically, strains with extremely low sequence similarity with the *P. agglomerans *type strain LMG 1286^T ^(well below 90%) were interspersed among better characterized Enterobacteriaceae.

Two plant epiphytic isolates (EPS486 and EPS595) from apple and pear trees and two wheat root isolates (P8SAA and P10QLC) formed a clearly distinct group in both trees which is not embraced by any of the MLST-groups defined for *Pantoea *[40], and are considered representative of a single new *Pantoea *spp.

**Figure 3:**
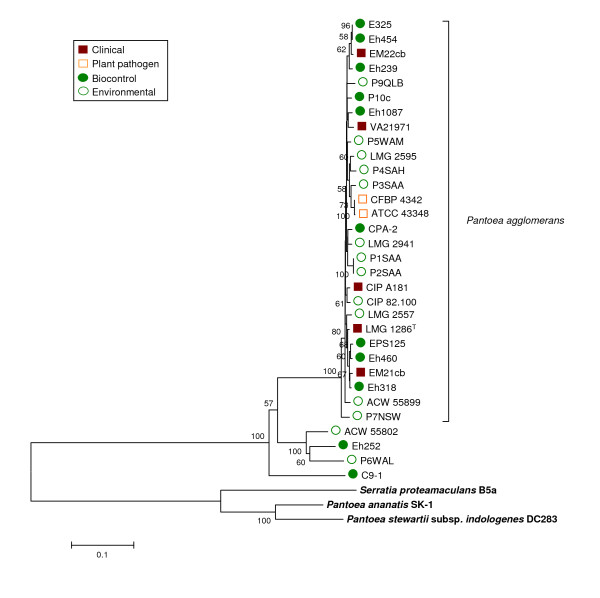
**Taxonomy of clinical and biocontrol P. agglomerans sensu stricto strains based on pagRI gene sequences**. The distance tree was generated with the Minimum Evolution method with the Maximum Composite Likelihood model using an 1168-bp fragment spanning the two genes. Sequences of autoinducer genes from related enterobacterial species P. stewartii pv. stewartii (genome project site http://www.hgsc.bcm.tmc.edu/microbial-detail.xsp?project_id=125), P. ananatis [GenBank accession AB304810] and S. proteamaculans [GenBank accession AY040209] were used as references. Nodal supports were assessed by 500 bootstrap replicates. Only bootstrap values greater than 50% are shown. The scale bar represents the number of substitutions per site.

The *P. agglomerans *AHL autoinducer encoding genes *pagRI *are located on a 530 kbp plasmid in the genome of strain C9-1 [42]. Amplification of primers designed for *pagRI *based on the C9-1 sequences was successful for all strains clustered with *P. agglomerans *type strain LMG 1286^T ^in the *rrs *tree independent of their ecological origin except strain LMG 5343. No amplification was observed for strains outside the *P. agglomerans sensu stricto *cluster. All strains positive with PCR were also positive for biosynthesis determined using the autoinducer biosensor. Notably, the only outlier strain, LMG 5343, does not cluster with *P. agglomerans *acccording to *gyrB *sequence analysis. The presence of *pagRI *matched the taxonomic clustering of *P. agglomerans *based upon *gyrB*, with a few strains (Eh252, ACW55802, P6WAL and C9-1) clustering independently from the type strain LMG 1286^T ^and without divergent grouping of clinical and biocontrol strains (Figure 3). Taxonomic determination of the subgroup containing strains Eh252, ACW55802 and P6WAL is ambiguous. All strains clustered with *P. agglomerans *type strain LMG 1286^T ^in the 16S rDNA tree, but were separated slightly from the main group both in the *gyrB*, *pagRI *and fAFLP trees, (as well as the reaction of AHL reporter strains) suggest that this group may constitute a new subspecies of *P. agglomerans*. Amplification of *pagRI *and AHL biosynthesis were positive for all *P. agglomerans *and no discrimination was observed among clinical or biocontrol isolates.

### fAFLP of P. agglomerans sensu stricto isolates

**Figure 4:**
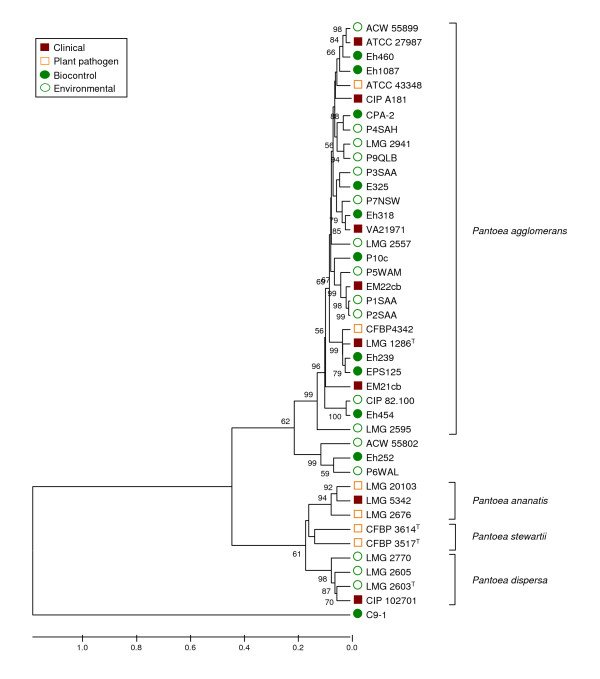
**Clustering of P. agglomerans sensu stricto strains based on UPGMA analysis of concatenated fAFLP patterns obtained using four selective primer combinations**. For each strain, a total of 885 data points (bands) indicating the presence (1) or absence (0) of an fAFLP peak were used in the analysis. P. ananatis, P. stewartii and P. dispersa strains were used as reference. Bootstrap values after 1000 replications are expressed as percentages. ^T^: type strain; LMG: Culture Collection, Laboratorium voor Microbiologie, Ghent, Belgium; CFBP: Collection Française de Bactéries Phytopathogènes INRA, Angers, France; CIP: Collection de l'Institut Pasteur, Paris, France; ATCC: American Type Culture Collection, Manassas VA, U.S.A; ACW: Agroscope Changins-Wädenswil, Wädenswil, Switzerland (own strains).

Analysis of fAFLP data was restricted primarily to strains identified as *P. agglomerans sensu stricto *by sequence analysis, with *P. ananatis*, *Pantoea stewartii *and *Pantoea dispersa *included in our analysis as outgroups. Four primer sets were used in the selective amplification step of fAFLP giving a pool of 885 possible peak positions, with an average of 103 peaks (signals) obtained for each strain. Each species formed a distinct cluster in the UPGMA dendrogram consistent with the arrangement of Brady et al. [43] and confirming the power of fAFLP as a tool for the molecular-based identification of *Pantoea *to the species level (Figure 4). The position of strain C9-1 was peculiar in so far that it clustered well outside the *P. agglomerans *group and showed almost no similarity even with other *Pantoea *spp., sharing only a very limited number of fAFLP peaks with other strains of the genus (Figure 4). These results were confirmed in three independent repetitions of the fAFLP analysis beginning with single colonies of each strain on different dates, and the identity of C9-1 DNA used in each fAFLP run was confirmed by *gyrB *sequencing. The fAFLP patterns were consistent with those from sequencing data (excepting C9-1), with a distinct *P. agglomerans sensu stricto *cluster and no separation of biocontrol and clinical strains within this group (Figure 4). This supports the redesignation of C9-1 into a new species, closely related to *P. agglomerans*.

**Figure 5:**
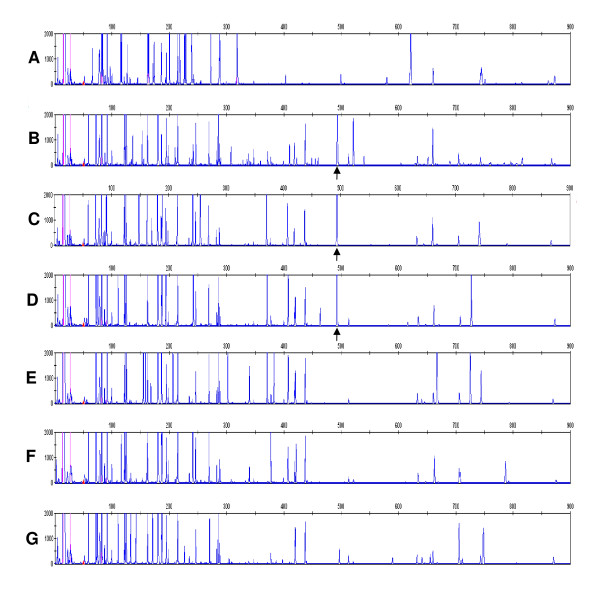
**The fAFLP pattern generated with EcoRI-G and MseI-G primers from different biocontrol, environmental and clinical P. agglomerans isolates**. (**A**) C9-1, (**B**) CIP 82.100, (**C**) P10c, (**D**) Eh325, (**E**) EM21cb, (**F**) EM22cb, (**G**) CIP A181. Biocontrol strain *P. agglomerans *C9-1 showed a completely divergent fAFLP pattern with respect to the other isolates of the species. The 490-bp band which was prevalent in biocontrol and environmental isolates, but was absent from clinical isolates and from strain C9-1 is indicated by the arrow.

Analyis of the fAFLP profiles failed to identify any peak(s) unique to clinical strains that could be used as marker for pathogenicity potential. However, a 474-bp band was obtained using EcoRI-G and MseI-G (+1) primers that was found in all plant isolates (biocontrol strains) but none of the clinical strains (Figure 5). The only exception was biocontrol strain C9-1 which lacked this 'biosafety' fAFLP band. Specific primers for the putative fAFLP 'biosafety' band were designed after cloning and sequencing the fragment. The band sequence consisted of a partial ORF identified as the encoding gene for a multidrug transport protein AcrF, which is part of a putative RND (resistance-nodulation-cell division type) efflux system. Primers for this gene amplified in both clinical and biocontrol strains, indicating that all strains carry this gene but that flanking regions may differ resulting in divergent fAFLP patterns.

### Comparison of other genotypic and phenotypic traits

**Figure 6:**
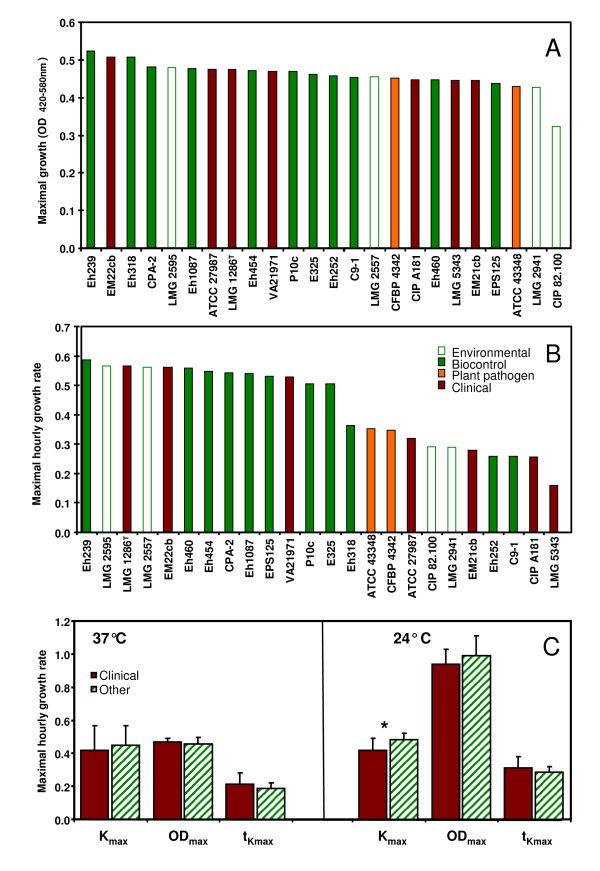
**Growth of Pantoea strains at 37°C and 24°C**. Maximal growth (**A**) and maximal hourly growth rate (**B**) of different isolates clustering with *P. agglomerans *LMG 1286^T ^in the *rrs *tree at 37°C. 0.25 OD_420-580 nm _units correspond to about 10^8 ^CFU/ml. The average values for maximal hourly growth rate (κ_max_) and maximal cell density (OD_max_) as well as the time needed to attain maximal hourly growth rate (t_kmax_, expressed in days) are shown in (**C**). The asterisk indicates a statistical difference (two-tailed t-test) between clinical and other isolates (i.e., environmental, biocontrol and plant pathogenic isolates).

Presence of traits that may reflect adaptation to the different lifestyles, such as sorbitol utilization, growth at 24°C and 37°C, and pantocin A or T3SS genes was determined in strains within the *P. agglomerans sensu stricto *cluster and the two most-closely related groups represented by strains Eh252 and C9-1. At 37°C none of these three investigated parameters were significantly different between presumptive-clinical and plant isolates [i.e., maximal cell density (OD_max_), maximal hourly growth rate (*k*_max_) and time needed to attain the maximal hourly growth rate (t_*k*max_)] (Figure 6). In fact, the maximal hourly growth rate was slightly less in clinical isolates, compared to *Pantoea *biocontrol or plant isolates. Similarly at 24°C, although clinical isolates had slightly lower maximal hourly growth rate compared to plant strains, differences were not significant (Figure 6). All strains of *P. agglomerans *grew poorly at 37°C compared to growth at 24°C.

Utilization of sorbitol by *P. agglomerans *as a sole carbon source was restricted to only a few biocontrol isolates, indicating this as an important feature for phytopathogen antagonism. In addition to the commercial biocontrol strain C9-1, which has two plasmid-encoded sorbitol-utilization operons [42], only the biocontrol strains Eh252 and P10c were able to efficiently metabolize sorbitol. Strain *P. ananatis *LMG 2665^T^, included as a positive control for sorbitol utilization, and *P. agglomerans *strains C9-1 and Eh252 gave absorbance readings that indicated a growth after 6-8 h from inoculation, while the lag-phase of P10c was protracted up to 24 h, suggesting that a certain signal may be required for this strain before C_6_-sugar metabolism is triggered.

Pantocin A biosynthetic genes were amplified in just four biocontrol isolates (i.e., C9-1, Eh252, Eh318 and CPA-2) and one clinical strain LMG 5343. Genome sequence analysis of C9-1 has revealed that in this strain the gene cluster coding for pantocine production is situated on a low-GC genomic island of about 29 kbp inserted between the *mutS *and *narL *genes, which was probably acquired by horizontal gene transfer [42]. Using primers narL-rev and mutS-rev, designed on the flanking regions of the insertion site, a 554-bp fragment could be amplified in all *P. agglomerans *strains that were negative for *paaABC *(i.e., with no genomic island insertion). The large size of the genomic island in *paaABC*-positive strains prevented recovery of a PCR-product amplicon. This indicates that the insertion site of the pantocin genomic island is the same as in C9-1 for all *Pantoea *strains carrying the pantocin A genes. The origin of the pantocin genes remains unknown, and no near or distant homologues have been identified in any other organism after an extensive BLAST search.

**Figure 7:**
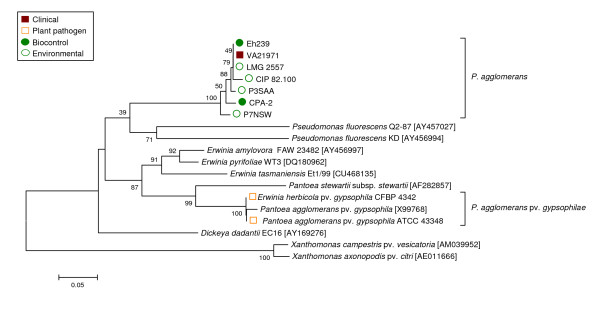
**Phylogeny of P. agglomerans sensu stricto strains of diverse origin based on partial sequencing the hrcN gene, coding for the type III secretion system-specific ATPase**. A total of 32 *P. agglomerans *or nearly related strains (e.g., SC-1 or LMG 5343) were tested for the presence of a T3SS using primers hrcN-4r and hrcN-5rR, which were designed on the basis of the alignment of the *hrcN *genes of *E. amylovora *and *Pseudomonas syringae*. A positive amplification was obtained as expected in two known plant pathogens (*P. agglomerans *pv. *gypsophilae *CFBP 4342 and *P. agglomerans *pv. *gypsophilae *ATCC 43348) and in seven more strains, including four environmental strains (CIP 82.100, LMG 2557, P3SAA and P7NSW), one clinical isolate (VA21971) and two biocontrol strains (Eh239 and CPA-2). Sequence analysis revealed that the *hrcN *gene found in the latter seven strains is more similar to that of biocontrol *P. fluorescens *and is not closely related to *P. agglomerans *pv. *gypsophilae *or other plant pathogenic bacteria, indicating a divergent function. GenBank accession numbers of reference sequences not obtained in this work are indicated between square brackets.

The T3SS gene *hrcN *was identified in several isolates (Figure 7), including the two phytopathogenic *P. agglomerans *pv. *gypsophilae *strains (i.e., ATCC 43348 and CFBP 4342) for which a T3SS has been previously reported [44,45]. Whether this suggests that these strains may have some T3SS components and thus have pathogenic potential (e.g., on plants) remains uncertain. Strains which amplified *hrcN *included four environmental strains (CIP 82.100, LMG 2557, P3SAA, P7NSW), one clinical strain (VA21971) and two biocontrol isolates (CPA-2, Eh239). Subsequent sequencing of the obtained fragment revealed that the *hrcN *gene in all of these strains diverged significantly from the *hrcN *sequence carried by *P. agglomerans *pv. *gypsophilae *and other plant pathogenic bacteria. The sequence they carried was more closely related to that of *Pseudomonas fluorescens *strains used in biocontrol of soilborne plant diseases (Figure 7), indicating a non-pathogenic alternative function.

## Discussion

Discrimination of clinical and plant-associated isolates of *P. agglomerans *has important implications for the registration of biocontrol products for plant protection. We conducted a polyphasic genotypic analysis of a collection of strains from different ecological niches. Our first observation was that a majority of clinical strains were in fact not true *P. agglomerans *as defined by Gavini et al. [1] based on taxonomic discrepencies revealed by sequence analysis of the 16S rDNA and *gyrB *genes. All biocontrol strains in the collection were found to be correctly identified as *P. agglomerans*. The reason for this discrepancy is ascribed to the fact that bacteria selected for their biological control properties are typically better characterized, including DNA sequencing, in comparison to those obtained in clinical diagnostics where rapid identification for implementation of therapeutic treatment is the primary concern and relies on less precise biochemical identification methods (e.g., API20E and Vitek-2 from bioMerieux or Phoenix from BD Diagnostic Systems). Biochemical methods have previously been shown to misidentify *P. agglomerans *and *Enterobacter *spp. [43,46-49], which our results confirm. Additionally, many archival strains were deposited in culture collections more than 30 years ago when the genus *Pantoea *was not yet taxonomically established and biochemical identification was less accurate.

**Figure 8:**
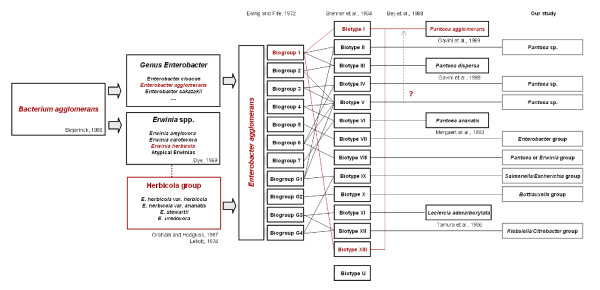
**Taxonomic rearrangements undergone by the E. agglomerans/E. herbicola complex in the last decades and attempts to assign still unassigned biotypes to known species**. Strains belonging to the *E. agglomerans*/*E. herbicola *complex were described as early as 1888 [59] and included organisms that were saprophytes or plant pathogens [60,61] or (opportunistic) pathogens in humans [61]. The name *E. agglomerans *was proposed by Ewing and Fife [50] after comparing plant and animal isolates as a subjective synonym for all three *Erwinia *species in the Herbicola group which was created in the meantime, i.e., *E. herbicola*, *E. stewartii *(now *P. stewartii*) and *E. uredvora *[62]. In this process, other *Enterobacter *strains may have been included in the new species. Brenner et al. [41] attempted to classify *E. agglomerans *strains by DNA hybridization and phenotypic tests deciding upon 13 biotypes. Subsequent classification efforts assigned several of the Brenner biotypes to new species, including *P. agglomerans*, *P. dispersa*, *P. ananatis *or *Leclercia adecarboxylata *[1,52,54], but for most reclassification with definitive assignment remains open. For these still unnamed biotypes an approximate classification, based on strain phylogeny (Figure 1 & 2) or 16S rDNA and *gyrB *sequence similarity (see Additional file 2- Table S2) is projected above.

The *Enterobacter/Pantoea *genus has undergone numerous taxonomical rearrangements [1,41,48,50-53] (Figure 8) and our results indicate that many strains previously identified as *E. agglomerans *or *E. herbicola *have been improperly transferred into the composite *P. agglomerans *species [1]. Although previous studies based on DNA-DNA hybridization alerted that the *E. agglomerans*-*E. herbicola *complex is composed from several unrelated species [52,54,55] (Figure 8), these names continue to be utilized as subjective synonyms. In this study, we analyzed the current subdivisions of *P. agglomerans *based on DNA-DNA hybridization and used sequence analysis to establish valid identity of representative strains for each *E. agglomerans *biotype as defined by Brenner et al. [41], and biotype XI *Leclercia adecarboxylata *[52]. We could not confirm the identity of strain LMG 5343 as *P. agglomerans*, indicating that biotype V should not be included in *P. agglomerans *as previously hypothesized by Beji et al. [53]. Our BLAST analysis of strains belonging to other biotypes that have not yet been assigned to a particular species showed the highest similarity of these strains to undefined *Enterobacter *or *Erwinia *spp. Sequences belonging to *P. agglomerans *isolates and a wide-range of other bacteria described as unknown or uncultured bacterium frequently were scattered as top hits in the BLAST-search (see Additional file 2 - **Table S2**). These sequences were not closely related to any of the individual type strains of the *Pantoea *species. This indicates the risk that a high number of *Enterobacter *and *Erwinia *strains present in the databases are misidentified as *Pantoea*. The problematic classification of strains belonging to the classical *E. agglomerans *basonym is further demonstrated by the observation of incorrect culture collection designations. In the CECT (Colección Española de Cultivos Tipo) three strains (including CECT 850, synonymous with the LMG 1286^T ^type strain) can be retrieved using "agglomerans" as search term. Strain CECT 4842, is retrievable as *E. herbicola *but listed as *P. agglomerans *despite the fact that our 16S rDNA data suggests this strain is *Klebsiella*, and strain CECT 842 received by us as *E. agglomerans *isolated from human feces has now been designated as the type strain of BL-2 *Cedecea davisae *[56]. In the BCCM/LMG collection (Belgian Co-ordinated Collections of Micro-organisms/Laboratorium voor Microbiologie, Universiteit Gent) many strains received as clinical *E. agglomerans *isolates are awaiting reclassification and are now considered "unidentified" (see Additional file 1 - Table S1). Most of the *P. agglomerans *strains obtained from the ATCC, particularly those of clinical origin, were found in our analysis to belong to other species. Thus, incorrect taxonomy is a major problem in terms of biosafety classification of *P. agglomerans*.

We identified a single discriminatory marker for biocontrol strains using fAFLP which may be of use in biosafety decisions for registration of beneficial isolates. Only biocontrol isolates had this fAFLP band, eventhough all strains of *P. agglomerans sensu stricto *have indication of the gene found within the band. For differentiation purposes this is irrelevant since the purpose is to identify a genomic marker, not a specific gene.

Our polyphasic analysis indicated that clinical and biocontrol strains co-cluster within *P. agglomerans sensu stricto*. This suggests that both isolates from clinical and environmental habitats have undergone indistinguishable evolutionary changes and that there is no discernable specialization of clinical isolates toward human pathogenicity or biocontrol isolates toward a plant-associated life-style. We observed no evidence, but can not exclude, the possibility that clinical isolates may have acquired specific pathogenicity factors beyond T3SS on plasmids or other mobile elements, as has been reported for phytopathogenic strains [44,45]. The T3SS discovered in some strains, however, was found to be more closely related to that in biocontrol *Pseudomonas *spp. indicating a non-pathogenic function [57]. Furthermore, only one clinical isolate had a T3SS gene compared to six environmental isolates. Comparison between the completed genome of biocontrol strain C9-1 and the in progress genome sequencing of the clinical type strain of *P. agglomerans *LMG 1286^T ^(T.H.M. Smits, B. Duffy et al., unpublished data) indicates that several features including antibiotic production (revealed by the presence of *paaABC *genes [58]), and nectar sugar utilization as a sole carbon source are generally associated with antagonistic activity. Our results demonstrate, however, that while many biocontrol strains have such traits, not all do and thus these are not universal features of biocontrol potential. Also, we have demonstrated for the first time the presence of the antibiotic biosynthetic genes *paaABC *in clinical strains, indicating that these may not be unique signatures of biocontrol isolates. What if any role pantocin may contribute to animal associations remains to be determined. There was no difference in growth at 37°C between clinical and biocontrol isolates, with both types of strains growing poorly at this temperature compared to growth at 27°C, and reinforcing the weakness of this criteria to determine pathogenicity.

Returning to the fundamental problem of insufficient confidence in identification procedures, we have shown that specific gene sequences (such as *gyrB *rather than 16S rDNA) are more robust than biochemical identification regarding *P. agglomerans*. The several reports of *P. agglomerans *from clinical literature upon which biosafety decisions have been based all lack a clear establishment of this species as a primary and singular cause of disease. With rare exception such isolates are not available for precise taxonomic confirmation and detailed clinical histories are typically absent for individual strains. We conducted a small survey of three clinical diagnostic laboratories in Switzerland and found that *P. agglomerans *is infrequently recovered. *P. agglomerans *was identified, predominantly as a polymicrobial co-isolate in patients, 21 times in the last four years at the ICM in Bellinzona (M. Tonolla, personal communication) and six times in the last three years at the Kantonsspital Lucerne (M. Hombach, personal communication). At the Institute for Medical Microbiology of Zürich University several strains identified as *Pantoea *were reported to be present in the collection of clinical samples, but no genetic sequence related to *P. agglomerans *was retrieved from their sequence database (G. Bloemberg, personal communication). Thus, *P. agglomerans *correctly characterized appears to be a more infrequent clinical organism than literature indicates.

## Conclusion

Our study indicates that current restrictions on registration of microbial pesticides based on *P. agglomerans *biocontrol strains in Europe warrant review. The primary argument for biosafety concerns is not supported by the fact that a majority of clinical strains are currently misclassified as *P. agglomerans *as determined by sequence analysis of 16S rDNA and *gyrB*. Further analysis of specific genes and fAFLP patterns also distinguish beneficial from clinical strains within *P. agglomerans sensu stricto*. Moreover, the lack of pathogenicity confirmatory tests with clinical strains (i.e., Koch's postulates) and the polymicrobial nature in clinical reports, which is probably just a reflection of the natural abundance of this species in the environment, draws into question the biosafety concerns with plant beneficial isolates.

## Authors' contributions

FR carried out the molecular genetic studies and phenotypic tests and drafted the manuscript. THMS participated in the design and the implementation of the phenotypic tests. EM isolated, characterized and provided strains, and contributed to the study design. JEF participated in the conception and execution of the study. BD conceived and led the study, and helped draft the manuscript. All authors read and approved the final manuscript.

## Supplementary Material

Additional file 1 **Table S1**. Strains used in this study (including references). Click here for file

Additional file 2 **Table S2**. BLAST hits obtained from NCBI blastn using 16S rDNA and *gyrB *sequences of representative strains belonging to the different *Enterobacter agglomerans *biotypes defined by Brenner et al. Click here for file
