# Supplementary material for: Genotypic comparison of Pantoea agglomerans plant and clinical strains
Source: BMC Microbiol. 2009 Sep 22;9:204. doi: 10.1186/1471-2180-9-204 (PMC2764716; doi:10.1186/1471-2180-9-204)
Supplement: Additional file 1 — Table S1. Strains used in this study (including references). [file 1471-2180-9-204-S1.pdf]

TABLE S1. Strains used in this study (ᵀ=type strain).

Strain designation	Received as	Origin	Host	Organ	Properties	Other designations, notes	References
Brenneria							
LMG 2694 T	*B. nigrifluens*	United States	*Juglans regia*	Bark canker	Phytopathogen	Type strain	[1]
LMG 2709 T	*B. rubrifaciens*	United States	*Juglans regia*	-	Phytopathogen	Type strain	[2]
Enterobacter							
LMG 2693T	*E. cancerogenus*	Czech Republic	*Populus canadensis* cv. *Regenerata*	-	Phytopathogen	Type strain	[3]
LMG 5336	*E. agglomerans*	United States	-	-	Clinical	*P. agglomerans* ATCC 29904	
LMG 5337	*E. agglomerans*	United States	Human	Intervenous fluid	Clinical	*P. agglomerans* ATCC 29915	
LMG 5339	*E. agglomerans*	United States	Chicken	Liver	Veterinarial	*P. agglomerans* ATCC 29917	
LMG 5341	*E. agglomerans*	United States	Human	-	Clinical	*P. agglomerans* ATCC 29919	
LMG 5345	*E. agglomerans*	United States	Human	-	Clinical	*P. agglomerans* ATCC 29923	
Erwinia							
CFBP 1232T	*E. amylovora*	United Kingdom	*Pyrus communis*	-	Phytopathogen	Type strain	[4]
CFBP 4342	*E. herbicola* pv. *gypsophilae*	Netherlands	*Gypsophila paniculata*	Plant gall	Phytopathogen		
DSM 12163 T	*E. pyrifoliae*	South Korea	*Pyrus pyrifolia*	-	Clinical	Type strain	[5]
Ea 273	*E. amylovora*	United States	*Malus pumila*	Apple	Phytopathogen	ATCC 49946	[6, 7]
Pantoea							
ACW 55802	*P. agglomerans*	Switzerland	Apple tree	-	Environmental		
ACW 55899	*P. agglomerans*	Switzerland	Apple tree	-	Environmental		
ATCC 27981	*P. agglomerans*	United States	Human	Sputum	Clinical	CDC 865-74	
ATCC 27982	*P. agglomerans*	United States	Human	IV fluid	Clinical	CDC 2214-72	
ATCC 27985	*P. agglomerans*	United States	Human	N/A	Clinical	CDC 811-75	
ATCC 27987	*P. agglomerans*	United States	Human	Ear	Clinical	CDC 1400-74	

ATCC 27988	*P. agglomerans*	United States	Human	Urine	Clinical	CDC 1377-71	
ATCC 27989	*P. agglomerans*	United States	Human	Sputum	Clinical	CDC 1300-74	
ATCC 27990	*P. agglomerans*	United States	Human	-	Clinical	CDC 1134-72	
ATCC 27991	*P. agglomerans*	United States	Human	Eye drainage	Clinical	CDC 358-74	
ATCC 27992	*P. agglomerans*	United States	Human	-	Clinical	CDC 1269-74	
ATCC 27993	*P. agglomerans*	United States	Human	Blood	Clinical	CDC 4-74	
ATCC 27994	*P. agglomerans*	United States	Human	-	Clinical	CDC 3072-74	
ATCC 27995	*P. agglomerans*	United States	Human	-	Clinical	CDC 4854-73	
ATCC 27996	*P. agglomerans*	United States	Insect	-	Environmental	CDC 5474-71	
ATCC 27998	*P. agglomerans*	United States	Human	Trachea	Clinical	CDC 1741-71	
ATCC 29001	*P. agglomerans*	United States	Human	Prostate	Clinical	CDC 164-75	
ATCC 29918	*P. agglomerans*	United States	Human	-	Clinical	CDC 4388-71	
C9-1	*P. agglomerans*	United States	*Malus malus*	Stem	Biocontrol	BlightBan C9-1™	[8]
CECT 4842	*P. agglomerans*	Sweden	Industrial	Paper mill process water	Environmental		
CIP 102343	*P. agglomerans*	Algeria	Human	Urine	Clinical		
CIP 102701	*P. agglomerans*	France	Human	Ear	Clinical		
CIP 82.100	*P. agglomerans*	Canada	Cereal plant	-	Environmental	ATCC 33243^{T}, type strain of *Erwinia herbicola*	[9]
CIP A181	*P. agglomerans*	France	Human	Blood	Clinical		
CPA-2	*P. agglomerans*	Spain	*Malus* sp.	Apple surface	Biocontrol		[10]
E325	*P. agglomerans*	United States	Apple tree	Blossoms	Biocontrol	BloomTime™	[11]
Eh1087	*P. agglomerans*	New Zealand	Apple tree	Blossom	Biocontrol	Produces phenazine	[12]
Eh239	*P. agglomerans*	United States	Barley plant	Kernels	Biocontrol		[13]
Eh252	*P. agglomerans*	United States	*Malus pumila*	-	Biocontrol		[14]
Eh318	*P. agglomerans*	United States	Apple tree	Stem	Biocontrol		[15]
Eh454	*P. agglomerans*	United States	Barley plant	Kernels	Biocontrol		[13]

Eh460	*P. agglomerans*	United States	Barley plant	Kernels	Biocontrol		[13]
EM13cb	*P. agglomerans*	Spain	Human	Blood	Clinical		
EM15cb	*P. agglomerans*	Spain	Human	Catheter	Clinical		
EM17cb	*P. agglomerans*	Spain	Human	Blood	Clinical		
EM21cb	*P. agglomerans*	Spain	Human	Bile	Clinical		
EM22cb	*P. agglomerans*	Spain	Human	Blood	Clinical		
EM2cb	*P. agglomerans*	Spain	Human	Urine	Clinical		
EPS486	*P. agglomerans*	Spain	Apple tree	Bud	Environmental		
EPS595	*P. agglomerans*	Spain	Pear tree	Bud	Environmental		
EPS125	*P. agglomerans*	Spain	Pear tree	Fruit	Biocontrol		[16]
LMG 1286^{T}	*P. agglomerans*	Zimbabwe	Human	Knee laceration	Clinical	Type strain: ATCC 27155^{T}, CECT 850^{T}	[17]
LMG 2557	*P. agglomerans*	United Kingdom	*Pyrus communis*	-	Environmental	NCPPB 1269	
LMG 2595	*P. agglomerans*	South Africa	*Allium cepa*	Necrotic stalk and leaf	Environmental		
LMG 2941	*P. agglomerans*	N/A	*Malus sylvestris*	Leaf surface	Environmental		
P10c	*P. agglomerans*	New Zealand	Apple	Flower	Biocontrol	BlossomBless™	[18]
P10Qld	*P. agglomerans*	Australia	Wheat plant	Roots	Environmental	QC1.38	
P1SA	*P. agglomerans*	Australia	Wheat plant	Roots	Environmental	PGP1.TCV14.2	
P2SA	*P. agglomerans*	Australia	Wheat plant	Roots	Environmental	PGP1.TCV14.6	
P3SA	*P. agglomerans*	Australia	Wheat plant	Roots	Environmental	SAwow11ct.3	
P4SA	*P. agglomerans*	Australia	Wheat plant	Roots	Environmental	SHj20.23	
P5WA	*P. agglomerans*	Australia	Wheat plant	Roots	Environmental	WMRst146.26	
P6WA	*P. agglomerans*	Australia	Wheat plant	Roots	Environmental	WLs185.22	
P7NSW	*P. agglomerans*	Australia	Wheat plant	Roots	Environmental	NMcc14.5	
P8SA	*P. agglomerans*	Australia	Wheat plant	Roots	Environmental	SAp2ww1.186	
P9Qld	*P. agglomerans*	Australia	Wheat plant	Roots	Environmental	QB1.20	
SC-1	*P. agglomerans*	United States	Cotton plant	-	Phytopathogen		[19]

VA21971	*P. agglomerans*	Switzerland	Human	Arm wound	Clinical		
ATCC 43348	*P. agglomerans pv. gypsophilae*	United States	*Gypsophila paniculata*	Plant gall	Phytopathogen	Crown gall disease	[20]
LMG 20103	*P. ananatis*	South Africa	Eucalyptus tree	-	Phytopathogen		
LMG 2665 ^{T}	*P. ananatis*	Brazil	*Ananas comosus*	-	Phytopathogen	Type strain	[21]
LMG 2676	*P. ananatis*	United States	*Puccinia graminis* f. sp. *tritici*	Uredia	Phytopathogen		[22]
LMG 5342	*P. ananatis*	United States	Human	-	Clinical	*P. ananatis* ATCC 29920	[21]
LMG 23359	*P. citrea*	Philippines	Pineapple plant	-	Environmental		[23]
LMG 2603	*P. dispersa*	Japan	Soil	-	Environmental		[17]
LMG 2605	*P. dispersa*	Tanzania	*Vigna unguiculata*	Seed	Environmental		
LMG 2770	*P. dispersa*	United States	Human	Blood culture	Clinical		
LMG 22097	*P. punctata*	Japan	Orange tree		Environmental		[23]
CFBP 3614 ^{T}	*P. stewartii* subsp. *indologenes*	India	*Setaria italica*	Leaf spot	Phytopathogen	Type strain, LMG 2632 ^{T}	[21, 24]
CFBP 3517 ^{T}	*P. stewartii* subsp. *stewartii*	United States	*Zea mays* var. *rugosa*	-	Phytopathogen	Type strain, LMG 2715 ^{T}	[21, 24]
DC283	*P. stewartii* subsp. *stewartii*	United States	*Zea mays* var. *rugosa*	-	Phytopathogen		[25]
CFBP 6632	*Pantoea oleae*	Spain	*Olea europaea*	Plant gall	Phytopathogen	Olive knot disease	
LMG 5343	*Pantoea* sp.	United States	Human	-	Clinical	*P. agglomerans* ATCC 29921	

References

1. Wilson EE, Starr MP, Berger JA: **Bark canker, a bacterial disease of the Persian walnut tree**. *Phytopathology* 1957, **47**:669-673.
2. Wilson EE, Zeitoun FM, Fredrickson DL: **Bacterial phloem canker, a new disease of Persian walnut trees.** *Phytopathology* 1967, **57**:618-621.
3. Schonheyder HC, Jensen KT, Frederiksen W: **Taxonomic notes: synonymy of *Enterobacter cancerogenus* (Urosevic 1966) Dickey and Zumoff 1988 and Enterobacter taylorae Farmer et al. 1985 and resolution of an ambiguity in the biochemical profile**. *Int J Syst Bacteriol* 1994, **44**:586-587.
4. Billing E, Baker LAE, Crosse JE, Garrett CME: **Characteristics of english isolates of *Erwinia amylovora* (Burrill) Winsow et al.** *J Appl Microbiol* 1961, **24**(2):195-211.
5. Kim WS, Gardan L, Rhim SL, Geider K: ***Erwinia pyrifoliae* sp. nov., a novel pathogen that affects Asian pear trees (*Pyrus pyrifolia* Nakai)**. *Int J Syst Bacteriol* 1999, **49**:899-906.
6. Bogdanove AJ, Bauer DW, Beer SV: **Erwinia amylovora secretes DspE, a pathogenicity factor and functional AvrE homolog, through the Hrp (type III secretion) pathway**. *J Bacteriol* 1998, **180**:2244–2247.
7. Bocsanczy AM, Beer SV, Perna NT, Biehl B, Glasner JD, Cartinhour SW, Schneider DJ, DeClerck GA, Sebaihia M, Parkhill J *et al*: **Contributions of the genome sequence of *Erwinia amylovora* to the fire blight community**. *Acta Hort* 2008, **793**:163-170.
8. Ishimaru CA, Klos EJ, Brubaker RR: **Multiple antibiotic production by *Erwinia herbicola***. *Phytopathology* 1988, **78**:746-750.
9. Kwon S-W, Go S-J, Kang H-W, Ryu J-C, Jo J-K: **Phylogenetic analysis of *Erwinia* species based on 16S rRNA gene sequences**. *Int J Syst Bacteriol* 1997, **47**(4):1061-1067.
10. Nunes C, Usall J, Teixidó N, Fons E, Viñas I: **Post-harvest biological control by *Pantoea agglomerans* (CPA-2) on Golden Delicious apples.** . *J Appl Microbiol* 2002, **92**:247-255.
11. Pusey PL, Stockwell VO, Rudell DR: **Antibiosis and acidification by *Pantoea agglomerans* strain E325 may contribute to suppression of *Erwinia amylovora***. *Phytopathology* 2008, **98**(10):1136-1143.
12. Kearns LP, Mahanty HK: **Antibiotic production by *Erwinia herbicola* Eh1087: its role in inhibition of *Erwinia amylovora* and partial characterization of antibiotic biosynthesis genes**. *Applied and environmental microbiology* 1998, **64**(5):1837-1844.
13. Braun-Kiewnick A, Jacobsen BJ, Sands DC: **Biological control of *Pseudomonas syringae* pv. *syringae*, the causative agent of basal kernel blight of barley, by antagonistic *Pantoea agglomerans***. *Phytopathology* 2000, **90**:368-375.
14. Vanneste JL, Yu J, Beer SV: **Role of antibiotic production by *Erwinia herbicola* Eh252 in biological control of *Erwinia amylovora***. *J Bacteriol* 1992, **174**(9):2785-2796.
15. Wright SAI, Zumoff CH, Schneider L, Beer SV: ***Pantoea agglomerans* strain EH318 produces two antibiotics that inhibit *Erwinia amylovora* in vitro**. *Applied and environmental microbiology* 2001, **67**(1):284-292.

16. Bonaterra A, Mari M, Casalini L, Montesinos E: **Biological control of *Monilinia laxa* and *Rhizopus stolonifer* in postharvest of stone fruit by *Pantoea agglomerans* EPS125 and putative mechanisms of antagonism**. *Int J Food Microbiol* 2003, **84**:93-104.
17. Gavini F, Mergaert J, Beji A, Mielcarek C, Izard D, Kersters K, De Ley J: **Transfer of *Enterobacter agglomerans* (Beijerinck 1888) Ewing and Fife 1972 to *Pantoea* gen. nov. as *Pantoea agglomerans* comb. nov. and description of *Pantoea dispersa* sp. nov.** *Int J Syst Bacteriol* 1989, **39**(3):337-345.
18. Vanneste JL, Cornish DC, Yu J, Voyle MD: **P10c: a new biological control agent for control of fire blight which can be sprayed or distributed using honey bees.** *Acta Hortic* 2002, **590**:231-235.
19. Medrano EG, Bell AA: **Role of *Pantoea agglomerans* in opportunistic bacterial seed and boll rot of cotton (*Gossypium hirsutum*) grown in the field**. *J Appl Microbiol* 2007, **102**:134-143.
20. Cooksey DA: **Galls of *Gypsophila paniculata* caused by *Erwinia herbicola*.** *Plant Dis* 1986, **70**:464-468.
21. Mergaert J, Verdonck L, Kersters K: **Transfer of *Erwinia ananas* (synonym, *Erwinia uredovora*) and *Erwinia stewartii* to the genus *Pantoea* emend. as *Pantoea ananas* (Serrano 1928) comb. nov. and *Pantoea stewartii* (Smith 1898) comb. nov., respectively, and description of *Pantoea stewartii* subsp. *indologenes* subsp. nov.** *Int J Syst Bacteriol* 1993, **43**(1):162-173.
22. Pon DS, Townsend CE, Wessman GE, Schmitt CG, Kingsolver CH: **A *Xanthomonas* parasitic on uredia of cereal rusts**. *Phytopathology* 1954, **44**:707-710.
23. Kageyama B, Nakae M, Yagi S, Sonoyama T: ***Pantoea punctata* sp. nov., *Pantoea citrea* sp. nov., and *Pantoea terrae* sp. nov. isolated from fruit and soil samples**. *Int J Syst Bacteriol* 1992, **42**(2):203-210.
24. Hauben L, Moore ERB, Vauterin L, Steenackers M, Mergaert J, Verdonck L, Swings J: **Phylogenetic position of phytopathogens within the *Enterobacteriaceae***. *Syst Appl Microbiol* 1998, **21**:384-397.
25. Coplin DL, Rowan RG, Chisholm DA, Whitmoyer RE: **Characterization of plasmids in *Erwinia stewartii*.** *Applied and environmental microbiology* 1981, **42**:599-604.
