# Supplementary material for: Genotypic comparison of Pantoea agglomerans plant and clinical strains
Source: BMC Microbiol. 2009 Sep 22;9:204. doi: 10.1186/1471-2180-9-204 (PMC2764716; doi:10.1186/1471-2180-9-204)
Supplement: Additional file 2 — Table S2. BLAST hits obtained from NCBI blastn using 16S rDNA and gyrB sequences of representative strains belonging to the different Enterobacter agglomerans biotypes defined by Brenner et al. [file 1471-2180-9-204-S2.pdf]

TABLE S2. BLAST hits obtained from NCBI blastn using 16S rDNA and *gyrB* sequences of representative strains belonging to the different *Enterobacter agglomerans* biotypes defined by Brenner et al. [1].

Biotype	Strain	16S rDNA, best reliable hit(s)[a]	Accession	% Match	*gyrB*, best reliable hit(s)[a]	Accession	% Match
I	LMG 1286	*Pantoea agglomerans* NCTC9381T	AJ251466.1	1505/1515 (99%)	*Pantoea agglomerans* LMG 2554	EF988811.1	733/741 (98%)
II	LMG 5345	*Erwinia tasmaniensis* ET1/99	CU468135.1	1438/1471 (97%)	*Pantoea* sp. LMG 24200<br>*Enterobacter sakazakii* BAA-894<br>*Erwinia tasmaniensis* ET1/99	EF988770.1<br>CP000783.1<br>NC_010694	664/745 (89%)<br>654/746 (87%)<br>629/740 (85%)
III	LMG 2605	*Pantoea dispersa* GTC 1472	AB273743.1	1460/1465 (99%)	*Pantoea dispersa* LMG 2603	EF988818.1	730/740 (98%)
IV	ATCC 27998	*Erwinia soli* Ew02<br>*Erwinia tasmaniensis strain* ET1/99	EF540891.1<br>CU468135.1	1404/1423 (98%)<br>1425/1470 (96%)	*Salmonella enterica* subsp. *enterica* sv. Schwarzengrund CVM19633	CP001127.1	628/724 (86%)
V	LMG 5343	*Pantoea agglomerans* LMG 2565	AF373196	1448/1476 (98%)	*Pantoea agglomerans* LMG 2565	EF988814.1	674/744 (90%)
VI	LMG 5342	*Pantoea ananatis* LMG 2665	Z96081	1343/1362 (98%)	*Pantoea ananatis* LMG 2665	EF988824.1	738/740 (99%)
VII	LMG 5336	*Enterobacter* sp. 638	CP000653	1442/1464 (98%)	*Enterobacter cloacae* ATCC 13047<br>*Citrobacter koseri* ATCC BAA-895	AB084011.1<br>CP000822.1	674/724 (93%)<br>661/722 (91%)
VIII	LMG 5341	*Erwinia persicinus*	AJ001190.1	1457/1464 (99%)	*Erwinia pyrifoliae* DSM12163	AB242885.1	672/740 (90%)
IX	ATCC 29918	*Escherichia hermannii* GTC 347<br>*Salmonella typhimurium* LT2	AB273738.1<br>AE008706.1	1451/1468 (98%)<br>1443/1467 (98%)	*Salmonella enterica* subsp. *enterica* sv. Heidelberg SL476	CP001120.1	668/737 (90%)
X	LMG 5339	*Buttiauxella agrestis* DSM 4586	AJ233400.1	1442/1449 (99%)	*Enterobacter sakazakii* ATCC BAA-894	CP000783.1	689/815 (84%)
XI	LMG 5338 [b]	-	-	-	-	-	-
XII	LMG 5337	*Klebsiella pneumoniae* MGH 78578<br>*Citrobacter koseri* ATCC BAA-895<br>*Salmonella enterica* subsp. *enterica* sv. Heidelberg SL476	CP000647.1<br>CP000822<br>CP001120	1424/1466 (97%)<br>1421/1465 (96%)<br>1420/1466 (96%)	*Citrobacter koseri* ATCC BAA-895<br>*Salmonella enterica* subsp. *arizonae*	CP000822.1<br>CP000880.1	674/743 (90%)<br>663/729 (90%)
XIII	EL107[c]	*Pantoea agglomerans* LMG 2565	AF373196.1	1466/1477 (99%)			

[a] Self-hits are excluded, only hits to established strains with accepted species name were considered. Hits complete sequenced organisms were preferred.

[b] No sequence available, but according to Tamura et al. [2] strains of biotype XI belongs to *Leclercia adecarboxylata*.

[c] 16S rDNA sequence of *P. agglomerans* EL107 was retrieved in the NCBI database under accession number FJ357815.

References

1. Brenner DJ, Fanning GR, Leete Knutson JK, Steigerwalt AG, Krichevsky MI: **Attempts to classify Herbicola group-*Enterobacter agglomerans* strains by deoxyribonucleic acid hybridization and phenotypic tests**. *Int J Syst Bacteriol* 1984, **34**(1):45-55.
2. Tamura K, Sakazaki R, Kosako Y, Yoshizaki E: ***Leclercia adecarboxylata* gen. nov., comb. nov., formerly known as *Escherichia adecarboxylata*.** *Curr Microbiol* 1986, **13**:179-184.
